# Metastatic myocardial abscess on the posterior wall of the left ventricle: a case report

**DOI:** 10.1186/1752-1947-2-258

**Published:** 2008-08-05

**Authors:** Javaid Iqbal, Iftikhar Ahmed, Wazir Baig

**Affiliations:** 1 Department of Cardiovascular Science, Queen's Medical Research Institute, University of Edinburgh, UK; 2 Department of Medicine, Scarborough General Hospital, North Yorkshire, UK; 3 Department of Cardiology, Leeds General Infirmary, West Yorkshire, UK

## Abstract

**Introduction:**

Myocardial abscess is a rare and potentially fatal condition. Metastatic myocardial abscess in the setting of infective endocarditis has been infrequently reported in the medical literature. To the best of the authors' knowledge no case of myocardial abscess affecting the free wall of the left ventricle secondary to infective endocarditis of a right-sided heart valve has been reported previously.

**Case presentation:**

We report a case of tricuspid valve endocarditis caused by *Staphylococcus aureus *and resulting in a myocardial abscess on the posterior wall of the left ventricle, far from the active valvular infection. We also briefly discuss the role of different investigation modalities including cardiac magnetic resonance imaging in diagnosing myocardial abscess.

**Conclusion:**

Myocardial abscess is a life-threatening illness. A high index of clinical suspicion is required to make a prompt diagnosis. Final diagnosis may need multi-modality imaging. An early diagnosis, aggressive medical therapy, multidisciplinary care and timely surgical intervention may save life in this otherwise fatal condition.

## Introduction

Myocardial abscess (MA) is a suppurative infection of the myocardium, endocardium, native or prosthetic valves, perivalvular structures or the cardiac conduction system. It is a potentially life-threatening disease, where early recognition and institution of appropriate medical and surgical therapy is vital for patient survival. The overall mortality rate associated with *Staphylococcus aureus *endocarditis is 42%. If treated with appropriate antibiotics and surgery, the mortality rate falls to 25%. The presence of an intracardiac abscess results in a 13.7-fold increase in mortality. In the past, most cases of MA were found during autopsy; however, detection of MA can now be achieved antemortem, using noninvasive diagnostic modalities including transthoracic echocardiography (TTE), transoesophageal echocardiography (TOE), radionuclide scintigraphy, computed tomography (CT) scan and cardiac magnetic resonance imaging (CMRI).

## Case presentation

A 28-year-old intravenous drug user was admitted in a district general hospital with a 2-week history of fever, malaise and myalgia. He had no past medical history of note. On examination he was pyrexial but haemodynamically stable. His cardiovascular examination revealed signs of tricuspid regurgitation. His respiratory, abdominal and neurological examination was normal. Clinically, the diagnosis of infective endocarditis (IE) was suspected. Three sets of blood cultures were drawn and empirical intravenous antibiotic treatment commenced.

His blood tests showed leukocytosis with predominant neutrophilia and mild normochormic, normocytic anaemia. His electrocardiogram revealed non-specific ST-changes but no conduction abnormality. His chest X-ray was unremarkable. TTE confirmed vegetations on the tricuspid valve with severe regurgitation. All other valves were normal. Blood cultures grew *S. aureus *and vigorous antibiotic treatment was continued appropriately. However, the patient's condition continued to deteriorate with spiking fever and raised inflammatory markers. He was referred to the regional cardiothoracic centre for evaluation of valve surgery in view of uncontrolled infection.

**Figure 1:**
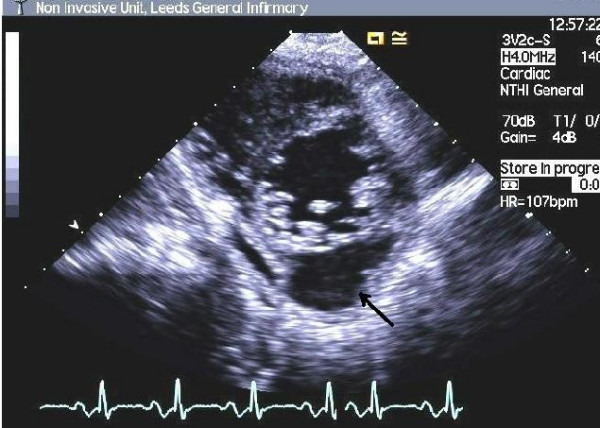
Transthoracic echo – Short axis view showing abscess cavity.

**Figure 2:**
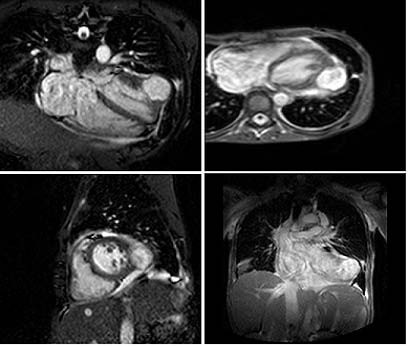
**Cardiac magnetic resonance imaging**. A 4.5 cm diameter left ventricular posterior wall abscess contained by only a 2 mm thin layer of myocardium.

On arrival at the cardiothoracic centre, the patient was acutely unwell with a temperature of 38.5°C, pulse of 120 beats per minute, blood pressure of 100/70 and respiratory rate of 26 breaths per minute. He had signs of severe tricuspid regurgitation and right heart failure. His repeat chest X-ray showed multiple cavitating lesions depicting metastatic pulmonary abscesses. There was also evidence of splenic abscesses on his abdominal ultrasound scan. Repeat TTE confirmed vegetations on the tricuspid valve with severe regurgitation but additionally it showed a small echo-free space in the wall of the left ventricle, raising suspicion of an MA (Figure 1). TOE was planned to evaluate this further but the patient was unable to tolerate it. Urgent CMRI was obtained, which revealed a 4.5 cm diameter left ventricular posterior wall abscess contained by only a 2 mm thin layer of myocardium (Figure 2 and Additional file 1). Urgent surgical intervention was planned but, unfortunately, the patient had a cardiac arrest prior to surgery and could not be resuscitated.

## Discussion

MA has been reported in about 20% of patients with IE [1]. They are usually adjacent to the area of valve infection and represent a direct extension of the infection [2]. Rarely, embolization of septic material results in a metastatic myocardial abscess remote from the main focus [3-6], as was the case here. The normal appearance of left-sided valves and concurrence of abscesses in extracardiac organs lead us to the conclusion that the left-sided MA had occurred as the result of embolization from the right-sided endocarditis.

Antemortem diagnosis of MA remains a challenge and a high index of clinical suspicion is required. TTE has a sensitivity of 23% and specificity of 98.6% in diagnosing MA [7]. At present, TOE is considered the investigation of choice but a recent prospective study of 115 patients revealed that it has only 48% sensitivity in diagnosing MA [8]. CMRI is a noninvasive imaging modality with high temporal and spatial resolution. To the best of our knowledge, no studies have compared the diagnostic value of TOE and CMRI in such cases. However, there are a few case reports and studies which suggest good diagnostic yield with CMRI in diagnosing annular abscess [9], sub-valvular abscess [10] and pseudo-aneurysm [11] in the setting of complicated IE. There may also be a complementary role for radionuclide imaging in diagnosing MA, where it can reveal a focal area of increased uptake in the myocardium suggesting the location of an abscess. It has low sensitivity but can be helpful in cases of prosthetic valve endocarditis where echocardiography may show too much scatter. Different radioisotopes including gallium-67, technetium-99 and indium-111 have been used in clinical practice with variable success [12,13].

Patients with this lethal disease can be saved by aggressive antibiotic treatment and prompt surgical intervention [5]. This can be best achieved by multidisciplinary care involving cardiologists, microbiologists, cardiac radiologists and cardiothoracic surgeons. Urgent surgery is recommended in most cases of MA since the perioperative risk and chances of rupture increase with the delay to surgery. However, the decision to perform emergency (same day) or urgent (1–2 days) surgery has to be made in individual cases depending on the clinical status of the patient, size of the abscess and thickness of the abscess wall. CMRI can provide useful morphological evaluation to help make this decision [10].

## Conclusion

In conclusion, MA is a life-threatening illness. A high index of clinical suspicion is required to make a prompt diagnosis. Final diagnosis may need multimodality imaging. Many of these patients may present to district hospitals where appropriate imaging and surgical facilities may not be available, and an urgent transfer to a specialist cardiothoracic centre is imperative. An early diagnosis, aggressive medical therapy, multidisciplinary care and timely surgical intervention may save the patient's life in this otherwise fatal condition.

## Abbreviations

CMRI: cardiac magnetic resonance imaging; IE: infective endocarditis; MA: myocardial abscess; TOE: transoesophageal echocardiography; TTE: transthoracic echocardiography.

## Competing interests

The authors declare that they have no competing interests.

## Authors' contributions

JI collected data, performed the literature search and drafted the manuscript. IA was involved in the literature search and manuscript review. WB supervised this patient's care and contributed to the preparation of the manuscript. All authors read and approved the final manuscript.

## Consent

Written informed consent was obtained from the patient's next of kin for publication of this case report and the accompanying images. A copy of the written consent is available for review by the Editor-in-Chief of this journal.

## Supplementary Material

Additional file 1 Cardiac magnetic resonance imaging of our patient showing morphological features of myocardial abscess. Click here for file
